# The relation between prescribing of different antibiotics and rates of mortality with sepsis in US adults

**DOI:** 10.1186/s12879-020-4901-7

**Published:** 2020-02-22

**Authors:** Edward Goldstein, Marc Lipsitch

**Affiliations:** 1 Center for Communicable Disease Dynamics, Department of Epidemiology, Harvard TH Chan School of Public Health, Boston, MA 02115 USA; 2 Department of Immunology and Infectious Diseases, Harvard TH Chan School of Public Health, Boston, MA 02115 USA

**Keywords:** Sepsis, Mortality, Antibiotics, Penicillins

## Abstract

**Background:**

Antibiotic use contributes to the rates of sepsis and the associated mortality, particularly through lack of clearance of resistant infections following antibiotic treatment. At the same time, there is limited information on the effects of prescribing of some antibiotics vs. others on subsequent sepsis and sepsis-related mortality.

**Methods:**

We used a multivariable mixed-effects model to relate state-specific rates of outpatient prescribing overall for oral fluoroquinolones, penicillins, macrolides, and cephalosporins between 2014 and 2015 to state-specific rates of mortality with sepsis (ICD-10 codes A40–41 present as either underlying or contributing causes of death on a death certificate) in different age groups of US adults between 2014 and 2015, adjusting for additional covariates and random effects associated with the ten US Health and Human Services (HHS) regions.

**Results:**

Increase in the rate of prescribing of oral penicillins by 1 annual dose per 1000 state residents was associated with increases in annual rates of mortality with sepsis of 0.95 (95% CI (0.02,1.88)) per 100,000 persons aged 75-84y, and of 2.97 (0.72,5.22) per 100,000 persons aged 85 + y. Additionally, the percent of individuals aged 50-64y lacking health insurance, as well as the percent of individuals aged 65-84y who are African-American were associated with rates of mortality with sepsis in the corresponding age groups.

**Conclusions:**

Our results suggest that prescribing of penicillins is associated with rates of mortality with sepsis in older US adults. Those results, as well as the related epidemiological data suggest that replacement of certain antibiotics, particularly penicillins in the treatment of different syndromes should be considered with the aim of reducing the rates of severe outcomes, including mortality related to bacterial infections.

## Background

Rates of hospitalization with a diagnostic code of septicemia or sepsis, associated mortality, as well as monetary costs of those hospitalizations have been rising rapidly during the past decades in the US [1–4]. A recent estimate from the US CDC suggests that about 270,000 Americans die annually as a result of sepsis [5]. Moreover, that estimate is expected to increase significantly if longer-term, e.g. 90-day mortality following sepsis diagnosis is accounted for [3]. Part of the reason behind the rapid growth in the rates of hospitalization with septicemia/sepsis in the diagnosis in the US is changes in diagnostic practices, including the implementation of sepsis screening protocols [6, 7]. However, changes in diagnostic practices in the US cannot fully explain the rise in the rates of hospitalization with septicemia/sepsis in the diagnosis, particularly prior to 2010 [8]. Indeed, trends in the rates of hospitalizations with any diagnosis of sepsis in the US between 2003 and 2009 closely resemble the trends in the rates of hospitalizations that involved infection and the use of mechanical ventilation [8]. Additionally, rates of hospitalization with severe sepsis in the diagnosis were growing robustly between 2008 and 2012, with the percent of hospitalizations with severe sepsis that involved multiple organ failure also rising during that period [9], suggesting genuine growth in the volume of hospitalization involving severe sepsis in the US. We also note that there is a long-term decline in the rates of healthcare-associated infections in the US [10, 11], suggesting that other types of infection are contributing to the growth in the volume of sepsis in the US.

Antibiotic use and resistance can contribute to the rates of sepsis hospitalization and mortality through several mechanisms, particularly lack of clearance of resistant infections/colonization episodes following antibiotic treatment, with some of those infections subsequently devolving into sepsis and lethal outcomes [12–17]. Generally, the relationship between antibiotic resistance and sepsis mortality is multi-step. First, antibiotic resistant infections are more likely to lead to a hospitalization with sepsis [16, 17]. For example, prevalence of amoxicillin-clavulanate (co-amoxiclav) resistance in bacteremia outcomes in England, particularly *E. coli* bacteremia is very high [18, 19], e.g. more than twice as high as prevalence of co-amoxiclav resistance in *E. coli*-related urinary tract infections [18]. Use of co-amoxiclav [20] to treat a milder *E. coli* infection may disproportionately fail when that infection is co-amoxiclav resistant, and a proportion of those treatment failures may lead to bacteremia, while they would not have if the initial infection had been susceptible and thus successfully treated. Secondly, for patients who are already hospitalized with sepsis, antibiotic resistance is often a risk factor for mortality, e.g. [14, 15].

Antibiotic use is one of the factors that affect the prevalence of antibiotic resistance [21–25]. Moreover, the use of a given antibiotic may also contribute to the prevalence of resistance to other antibiotics through various mechanisms of cross-resistance [22, 26–28]. Correspondingly, the use of different antibiotics is expected to affect the rates of sepsis and sepsis-associated mortality by both propagating antibiotic resistance, and leading to sepsis and associated deaths when antibiotics are used against infections resistant to those antibiotics. Modeling studies suggest that community use of antibiotics plays a bigger role in the acquisition of resistant infections than the in-hospital use of antibiotics [29, 30], though in-hospital antibiotic prescribing may also be an important contributor to the propagation of resistant infections and the associated severe outcomes [14, 15]. At the same time, there is limited information in the literature about the relation between the use of different antibiotics, particularly antibiotic prescribing in the community, and the risk/rates of sepsis and the associated mortality. Our earlier work [12] studied the relation between the outpatient prescribing of different antibiotics and rates of septicemia hospitalization in US adults. In this paper, we adopt a similar framework to the one used in [12] to examine the relation between outpatient prescribing of four major antibiotic classes (fluoroquinolones, penicillins, cephalosporins and macrolides) and the rates of mortality with sepsis in each of several age groups of US adults. Those analyses are based on the state-level US CDC Antibiotic Resistance Patient Safety Atlas data on outpatient antibiotic prescribing [31] and US CDC data on mortality [32] between 2014 and 2015. We hope that such ecological analyses would lead to further work on the effect of antibiotic prescribing, including evaluating replacement of some antibiotics by others and reduction in antibiotic prescribing on the rates of bacteremia, sepsis and associated mortality.

## Methods

### Data

Data on annual state-specific mortality with sepsis (ICD-10 codes A40-A41.xx representing either the underlying or a contributing cause of death) between 2014 and 2015 for different age groups of adults (18-49y, 50-64y, 65-74y, 75-84y, 85 + y) were extracted from the US CDC Wonder database [32]. For each age group, those data are available for the 50 US states and the District of Columbia (sample size of 51). Around 81.2% of deaths with sepsis in US adults aged over 18y between 2014 and 2015 (our study period) took place in the inpatient setting, with another 3% of those deaths taking place in the outpatient/ER setting. Additionally, for most of those deaths, sepsis is listed as a contributing rather than the underlying cause of death on the death certificate [33]. Among the annual average 180,908 deaths with sepsis in US adults aged over 18y between 2014 and 2015, only 37,993 (21%) had sepsis listed as the underlying cause of death. We believe that including all deaths with sepsis listed on the death certificate rather than just deaths with sepsis listed as the underlying cause better represents the volume of sepsis-related mortality in the US. Indeed, the relatively infrequent listing of sepsis as the underlying cause of death is likely related to coding practices for sepsis on mortality records, with infections [34] underreported as the proximal cause of death. Moreover, evidence from New York City suggests that upon additional training for the mortality coding personnel, the proportion of deaths with sepsis as the underlying cause increases significantly [35]. Therefore among the deaths with sepsis in US adults considered in this study, the number of those for which sepsis is the principal contributor is likely higher that the 21% of deaths which have sepsis listed as the underlying cause of death. Moreover, a recent study of US deaths meeting the clinical criteria of sepsis [36] tells that for 198/300 (66%) of those deaths, sepsis was the immediate cause of death. Those considerations, as well as the current US CDC estimate of 270,000 annual deaths as a result of sepsis [5] suggest that deaths having sepsis listed on the death certificate rather than only deaths having sepsis as the underlying cause of death better represent the volume of sepsis-related mortality in the US.

Data on the annual state-specific per capita rates of outpatient prescribing for four classes of oral antibiotics: fluoroquinolones, penicillins, macrolides, and cephalosporins, as well as overall antibiotic prescribing in different states in 2014 and 2015 were obtained from the US CDC Antibiotic Patient Safety Atlas database [31]. Annual state-specific population estimates in each age group of adults (overall, as well as the number of African-Americans) were obtained as the yearly July 1 population estimates in [37]. Data on median household income for US states between 2014 and 2015 were extracted from [38]. Data on average daily temperature for US states were obtained from [39]. Data on the percent of state residents in different age groups who lacked health insurance were extracted form the US Census Bureau database [40].

### Statistical inference

We apply a mixed-effect multivariable model to adjust for various factors that affect the relation between prescribing of different antibiotics and rates of severe outcomes (including mortality) associated with bacterial infections. For each age group of adults, (18-49y, 50-64y, 65-74y, 75-84y, 85 + y), we applied mixed effect models to relate the average annual state-specific outpatient prescribing rates (per 1000 state residents) for oral fluoroquinolones, penicillins, macrolides, and cephalosporins between 2014 and 2015 to the average annual state-specific rates of mortality with sepsis per 100,000 individuals in a given age group between 2014 and 2015 (dependent variable). Besides the antibiotic prescribing rates, the other covariates were the state-specific median household income, percentages of state residents in a given age group who were African American, those who lacked health insurance (in the non-elderly age groups, as health insurance, particularly Medicare coverage levels in the elderly are very high), as well as the state-specific average annual temperature. We note that sepsis mortality rates in African Americans are higher than in the overall population [41]. We also note that temperature may influence bacterial growth rates and/or transmission mediated effects [42], which in turn may affect both the prevalence of antibiotic resistance [42, 43], and the acquisition/severity of bacterial infections. To adjust for additional factors not accounted for by the covariates used in the model, we include random effects for the ten Health and Human Services (HHS) regions in the US. Specifically, for each state *s*, let *MR*(*s*) be the average annual state-specific rate of mortality (per 100,000) with sepsis in the *given age group* between 2014 and 2015, *A*_*i*_(*s*) (*i* = 1, .., 4) be the average annual state-specific outpatient prescribing rates, per 1000 state residents (of all ages), for the four studied classes of antibiotics between 2014 and 2015 (thus *A*_1_(*s*) denotes the rate of prescribing of oral fluoroquinolones, etc.); *I*(*s*) be the median state-specific household income between 2014 and 2015; *T*(*s*) be the state-specific average annual temperature (°*F*) between 2002 and 2011; *AA*(*s*) be the age-specific percent of state residents between 2014 and 2015 who were African American; *LHI*(*s*) be the average annual age-specific percent of state residents who lacked health insurance between 2014 and 2015 (for non-elderly age groups); *α*(*s*) be the random effect for the corresponding HHS region, and *ε* be the residual. Then

1 $$ MR(s)={\beta}_0+\sum \limits_{i=1}^4{\beta}_i\bullet {A}_i(s)+{\beta}_5\bullet I(s)+{\beta}_6\bullet T(s)+{\beta}_7\bullet AA(s)+ $$

$$ +{\beta}_8\bullet LHI(s)+\alpha (s)+\varepsilon\ (1) $$

## Results

Table 1 shows, for each age group of adults, the mean (standard deviation) for the state-specific average annual rates of mortality with sepsis per 100,000 individuals in that age group between 2014 and 2015, as well as the linear correlation between those rates and state-specific rates of outpatient prescribing of all oral antibiotics. The latter correlations are high, ranging from 0.59(0.37,0.74) for ages 85 + y to 0.77(0.62,0.68) for ages 65-74y. Additionally, annual rates of mortality with sepsis increase rapidly with age, from the state-specific mean of 8.31/100,000 for persons aged 18-49y to a mean of 750/100,000 for persons aged 85 + y.

**Table 1:** Rates of mortality with sepsis in the US and correlations with antibiotic prescribing rates

	Mean (standard deviation)	Linear correlation with rate of prescribing of all oral antibiotics
Rate of mortality with sepsis, ages 18-49y	8.31 (2.98)	**0.66 (0.47,0.79)**
Rate of mortality with sepsis, ages 50-64y	55.8 (17.3)	**0.74 (0.59,0.84)**
Rate of mortality with sepsis, ages 65-74y	143.4 (36.5)	**0.77 (0.62,0.68)**
Rate of mortality with sepsis, ages 75-84y	330.8 (75.6)	**0.67 (0.48,0.80)**
Rate of mortality with sepsis, ages 85 + y	750 (161.9)	**0.59 (0.37,0.74)**

State-specific rates of mortality with sepsis (ICD-10 codes A40–41.xx present as either underlying or contributing causes on a death certificate) per 100,000 individuals in different age groups between 2014 and 2015 (mean + standard deviation), and the linear correlation between those rates and state-specific rates of outpatient prescribing of all oral antibiotics

Table 2 shows the results of the multivariable model given by eq. 1. Table 2 suggests positive associations (with largest effect size in the corresponding age groups) between rates of outpatient prescribing of oral penicillins and rates of mortality with sepsis in individuals aged 75-84y and 85 + y. Increase in the rate of prescribing of oral penicillins by 1 annual dose per 1000 state residents was associated with increases in annual rates of mortality with sepsis of 0.95 (95% CI (0.02,1.88)) per 100,000 persons aged 75-84y, and of 2.97 (0.72,5.22) per 100,000 persons aged 85 + y. Table 2 also shows a negative association between rates of outpatient prescribing of oral penicillins and rates of mortality with sepsis in individuals aged 18-49y, and a positive association between the rates of outpatient prescribing of oral cephalosporins and rates of mortality with sepsis in individuals aged 18-49y (with effect sizes in the 18-49y age group being notably smaller than the effect sizes in the older age groups). Additionally, Table 2 shows positive associations between the percent of individuals aged 50-64y lacking health insurance, as well as the percent of individuals aged 65-74y and 75-84y who were African-American and rates of mortality with sepsis.

**Table 2:** Associations between different factors and US rates of mortality with sepsis in a multivariable model

	Aged 18-49y	Aged 50-64y	Aged 65-74y	Aged 75-84y	Aged 85 + y
Fluoroquinolones (prescription per 1000 residents/y)	0.01 (− 0.04,0.07)	0.15 (− 0.15,0.45)	0.26 (− 0.4,0.92)	− 0.16 (− 1.77,1.45)	− 0.61 (− 4.52,3.3)
Penicillins (prescription per 1000 residents/y)	− 0.03 (− 0.07,0)	0.08 (− 0.1,0.25)	0.11 (− 0.28,0.5)	**0.95 (0.02,1.88)**	**2.97 (0.72,5.22)**
Cephalosporins (prescription per 1000 residents/y)	**0.05 (0.02,0.09)**	0.07 (− 0.11,0.25)	0.13 (− 0.28,0.55)	−0.06 (− 1.04,0.93)	−0.76 (− 3.09,1.58)
Macrolides (prescription per 1000 residents/y)	0.02 (− 0.02,0.06)	0.06 (− 0.15,0.26)	0.21 (− 0.26,0.69)	0.45 (− 0.69,1.58)	0.71 (−2.03,3.45)
Median household income ($1000)	−0.06 (− 0.13,0.01)	−0.17 (− 0.55,0.2)	−0.09 (− 0.9,0.73)	0.54 (− 1.4,2.49)	1.95 (− 2.7,6.59)
Average minimal daily temperature (°*F*)	− 0.04 (− 0.11,0.03)	0.16 (− 0.19,0.51)	0.33 (− 0.44,1.1)	0.6 (− 1.26,2.46)	4.07 (− 0.55,8.7)
Percent African Americans	0.03 (− 0.05,0.11)	0.28 (−0.06,0.63)	**1.25 (0.41,2.1)**	**2.68 (0.76,4.6)**	3.02 (− 1.63,7.66)
Percent lacking health insurance	0.05 (−0.1,0.2)	**0.95 (0.01,1.89)**	ND	ND	ND

Regression coefficients for the different covariates in the model given by eq. 1 for different age groups. The coefficients for the different antibiotic classes estimate the change in the annual rates of mortality with sepsis (per 10,000 individuals in a given age group) when the annual rate of outpatient prescribing of oral antibiotics in the corresponding class (per 1000 residents) increases by 1. ND = not done because persons aged > 64 years old are eligible for Medicare

## Discussion

Rates of mortality related to septicemia/sepsis in the US are high [1–3, 5], and antimicrobial use may affect those rates through a variety of mechanisms (Introduction). However, our understanding of the effect of the use of certain antibiotics vs. others for various indications on the rates of sepsis and associated mortality is still limited. In this paper, we relate the proportions of different antibiotic types/classes among the overall volume of outpatient antibiotic prescription in different US states [31] to rates of mortality with sepsis in different age groups of US adults [32]. Our results suggest that prescribing of penicillins is associated with rates of mortality with sepsis in older US adults, with that finding supporting our earlier results on the association between the use of penicillins and rates of septicemia hospitalization in older US adults [12]. We also note the high prevalence of resistance to penicillins in both the Gram-negative and Gram-positive infections [44–46]. Additionally, our analyses suggest a positive association between the percent of individuals lacking health insurance and rates of mortality with sepsis in persons aged 50-64y, as well as the percent of individuals who are African-American and rates of mortality with sepsis in persons aged 65-84y, supporting the fact that rates of mortality with sepsis in African Americans are elevated [41, 47]. While our results lend support for the replacement of penicillins by other antibiotics with the aim of reducing the rates of sepsis and associated mortality, more granular analyses, particularly individual-level studies relating prescribing of different antibiotics in the treatment of a given syndrome to subsequent outcomes are needed to inform guidelines for antibiotic, particularly penicillin replacement, as well as for reduction in antibiotic prescribing, as explained further in the next two paragraphs.

Our findings about the positive associations between the use of penicillins and mortality with sepsis in older US adults are in agreement with the fact that prevalence of resistance to penicillins, particularly in older adults, is high for a variety of infections with both Gram-negative and Gram-positive bacteria [44–46]. Prevalence of resistance to fluoroquinolones for certain infections is also high (e.g. [44, 48]). However, multivariable analyses shows no evidence for the relation between prescribing of fluoroquinolones and mortality with sepsis (Table 2). Macrolides, while used relatively infrequently in the treatment of urinary tract and gastrointestinal infections, are commonly prescribed in the treatment of other sources of sepsis, particularly respiratory illness, both chronic [49] and acute, including pneumonia [50], with high prevalence of macrolide resistance in the corresponding infections [51]. At the same time, it was suggested that the use of non-lytic antibiotics, particularly macrolides in the treatment of lower respiratory tract infections (LRTIs) may decrease the likelihood of adverse outcomes [52, 53]. The positive point estimates for the association between macrolide prescribing and rates of mortality with sepsis in older adults fail to reach statistical significance in the multivariable model (Table 2). Finally, we found an association between prescribing of cephalosporins and rates of mortality with sepsis in adults aged 18-49y; however, no such associations were found in adults aged over 50y (Table 2). Lack of an association between prescribing of cephalosporins and mortality with sepsis in adults aged over 50y may be related to the fact that prevalence of resistance to cephalosporins in older adults is quite lower than prevalence of resistance to certain other types of antibiotics, particularly penicillins and fluoroquinolones, e.g. [44]. At the same time, prevalence of cephalosporin resistance and the frequency of extended-spectrum beta-lactamase (ESBL) production, including in Gram-negative bacteria is growing [54], and replacement of other antibiotics by cephalosporins might potentially lead to negative effects in the long run.

Our findings bring about the question regarding the relative utility of antibiotic replacement vs. reduction in antibiotic prescribing for reducing the rates of sepsis and the associated mortality. A key mechanism relating antibiotic use to the rates of severe outcomes associated with bacterial infection is lack of clearance of resistant infections following antibiotic treatment, with some of those infections subsequently devolving into bacteremia/sepsis and lethal outcomes. While replacement of antibiotics (particularly penicillins) by those to which prevalence of resistance is lower should decrease the scale of this phenomenon, reduction in antibiotic prescribing without antibiotic replacement is not expected to bring down the rates of severe outcomes associated with bacterial infections, at least in the short term, as no treatment should generally be worse compared to antibiotic treatment with regard to sepsis-related outcomes. We note that rates of bacteremia kept growing rapidly in England [18] while the rates of antibiotic consumption in the UK dropped by 7.3% from 2014 to 2017 [55]. Reduction in antibiotic prescribing may contribute to decreases in the rates of severe outcomes associated with bacterial infections in the longer term through decreases in antibiotic resistance. While overall recommendations for reduction in antibiotic use are commonly issued by public health entities in different countries, e.g. [55], recommendations for replacement of certain antibiotics by certain others in the treatment of certain syndromes (like the recommendation for the replacement of trimethoprim by nitrofurantoin in England, [19] p. 6) are generally less common. Such recommendations related to penicillin use (e.g. in-hospital co-amoxiclav prescribing in England) should have a notable effect on the rates of bacteremia/sepsis and associated mortality, both in England and the US. Finally, we note that recently, US FDA has recommended the restriction of fluoroquinolone use for certain conditions (such as uncomplicated UTIs) due to potential adverse effects [56]. However, no indications for antibiotics serving as replacement of fluoroquinolones were suggested in the FDA guidelines [56]. Such indications are needed to optimize the effect of those guidelines on the rates of severe outcomes associated with bacterial infections rather than possibly contribute to increases in the rates of such outcomes (e.g. increases in the rates of septicemia/sepsis and associated mortality through increases in the prescribing of penicillins).

Our paper has some limitations. Associations between prescribing of different antibiotic classes (particularly penicillins) and rates of mortality with sepsis may be affected not only by the contribution of different antibiotics to the rates of mortality with sepsis but also by patterns of antibiotic prescribing in different locations. We note that penicillins are prescribed for a wide variety of indications in the US, affecting prevalence of infection/colonization with different bacterial pathogens (e.g. [22]), and that there is high prevalence of resistance to penicillins in both the Gram-negative and Gram-positive bacteria in the US (e.g. [44–46]), all of which supports our results about the relation between prescribing of penicillins and rates of severe mortality with sepsis. The antibiotic-sepsis mortality associations that we found in the multivariable model estimate causal effects only if the model is well-specified and all confounders are accounted for in the analysis. One potential important source of confounding is the in-hospital antibiotic use. While there is evidence that community prescribing of antibiotics plays a bigger role than in-hospital prescribing in the acquisition of antibiotic resistance [29, 30], various synergistic effects between community and in-hospital antibiotic prescribing take place, both in terms of promoting the prevalence of resistance as well as the progression to sepsis, with some infections not cleared by the community use of antibiotics leading to hospitalizations, and the subsequent hospital use of antibiotics leading to sepsis and lethal outcomes. To adjust for potential effects of unmeasured confounding (including in-hospital antibiotic use) and residual confounding, we included random effects for the ten US Health and Human Services regions, which led to an improvement in the model fits. Further work involving more granular data, particularly individual-level analysis relating prescribing of different antibiotics in the treatment of a given syndrome to subsequent outcomes is needed to better ascertain the strength of the associations found in this paper. Coding practices for sepsis on death certificates may vary by US state [35]. Additionally, data on outpatient antibiotic prescribing in the whole population [31] were related to age-specific rates of mortality with sepsis in the US [32]. We expect that those sources of noise/incompatibility should generally reduce precision and bias the correlations towards null rather than create spurious associations.

## Conclusions

We believe that despite some limitations, our results suggest that prescribing of certain antibiotics, particularly penicillins is associated with rates of mortality with sepsis in older US adults, with the latter result supporting our earlier findings about the association between the rates of prescribing of penicillins and rates of hospitalization with septicemia in older US adults [12]. Additionally, there is high prevalence of resistance to penicillins for a variety of bacterial infections [44–46]. While these findings support the potential utility of replacement of penicillins by other antibiotics with the goal of reducing the rates of sepsis and associated mortality, further studies, including individual-level analyses are needed to better understand the effect of replacement of certain antibiotics, particularly penicillins by other antibiotics in the treatment of different syndromes, well as the effect of reduction in antibiotic prescribing in the treatment of certain conditions on the rates of severe outcomes associated with bacterial infections.

## Data Availability

The datasets generated and/or analysed during the current study are available in the following repositories: 1. US CDC. Antibiotic Resistance Patient Safety Atlas. Outpatient Antibiotic Prescription Data. https://gis.cdc.gov/grasp/PSA/indexAU.html 2. US CDC Wonder. Multiple Cause of Death, 1999–2017 Request. https://wonder.cdc.gov/mcd-icd10.html 3. US CDC. Bridged-Race Population Estimates, 1990–2017 data request. https://wonder.cdc.gov/Bridged-Race-v2017.HTML 4. United States Census Bureau. American FactFinder. Median Household Income. 2019. https://factfinder.census.gov/faces/nav/jsf/pages/index.xhtml 5. US CDC Wonder. North America Land Data Assimilation System (NLDAS) Daily Air Temperatures and Heat Index (1979–2011) Request. https://wonder.cdc.gov/nasa-nldas.html 6. US Census Bureau. Current Population Survey (CPS) (2018). CPS Table Creator. https://www.census.gov/cps/data/cpstablecreator.html

## Abbreviations

*E. coli*: *Escherichia coli*
US CDC: United States Centers for Disease Control and Prevention
UTI: Urinary tract infection
